# PET imaging of chemokine receptor CXCR4 in patients with primary and recurrent breast carcinoma

**DOI:** 10.1186/s13550-018-0442-0

**Published:** 2018-09-06

**Authors:** Tibor Vag, Katja Steiger, Andreas Rossmann, Ulrich Keller, Aurelia Noske, Peter Herhaus, Johannes Ettl, Markus Niemeyer, Hans-Jürgen Wester, Markus Schwaiger

**Affiliations:** 1 Clinic of Nuclear Medicine, Klinikum Rechts der Isar, Technische Universität München, Ismaninger Strasse 22, 81675 Munich, Germany; 2 Institute of Pathology, Technische Universität München, Troger Strasse 18, 81675 Munich, Germany; 3 III Medical Department, Klinikum Rechts der Isar, Technische Universität München, Ismaninger Strasse 22, 81675 Munich, Germany; 4 Clinic of Gynecology, Klinikum Rechts der Isar, Technische Universität München, Ismaninger Strasse 22, 81675 Munich, Germany; 5 Pharmaceutical Radiochemistry, Technische Universität München, Walther-Meissner Strasse 3, 85748 Garching, Germany

**Keywords:** CXCR4, Chemokine receptor, Positron emission tomography, Breast cancer

## Abstract

**Background:**

CXCR4 is a chemokine receptor frequently overexpressed in invasive breast cancer that has been shown to play a major role in signaling pathways involved in metastasis. The aim of this retrospective analysis was to assess the diagnostic performance of CXCR4-directed PET imaging in patients with breast cancer using the recently introduced CXCR4-targeted PET probe ^68^Ga-Pentixafor.

**Results:**

Thirteen patients with first diagnosis of breast cancer, four patients with recurrent disease after primary breast cancer, and one patient with axillary lymph node metastasis of unknown primary underwent CXCR4-targeted PET imaging using ^68^Ga-Pentixafor. Maximum standardized uptake values (SUVmax) and tumor-to-background (T/B) ratios of tumor lesions were measured and compared with pathological prognostic factors and molecular subtypes. ^18^F-FDG PET/CT images were available in 8/18 cases and were compared semi-quantitatively. Comparison with CXCR4 expression determined by immunohistochemistry was performed in 7/18 patients.

Nine of 13 primary breast cancers were visually detectable on ^68^Ga-Pentixafor PET images (mean SUVmax of 3.0). The visually undetectable lesions included both cases of invasive lobular carcinoma (ILC) and two cases of invasive carcinoma of no special type (NST) without any hormone receptor and HER2 expression (triple negative). Metastases of recurrent breast cancer and unknown primary cancer were visually detectable in all five cases, exhibiting a mean SUVmax of 3.5. ^18^F-FDG PET demonstrated higher SUVmax in all patients compared to ^68^Ga-Pentixafor PET. A correlation between SUVmax obtained from ^68^Ga-Pentixafor PET and prognostic factors including estrogen receptor (ER), progesterone receptor (PR), human epidermal growth factor receptor 2 (HER2) status, proliferation index, tumor grade, or molecular subtypes was not observed.

**Conclusions:**

CXCR4-directed PET imaging in patients with primary and recurrent breast cancer is feasible; however, tumor detectability is significantly lower compared to ^18^F-FDG PET. Moreover, we did not find any correlation between aforementioned prognostic factors of breast cancer and CXCR4-targeted tracer accumulation. Based on these results in a small patient cohort, CXCR4-targeted PET imaging does not seem to be suitable as a general diagnostic tool for imaging of breast cancer. Future CXCR4 imaging studies should investigate whether this modality might be useful in more specific applications, e.g., in therapeutic approaches especially under the view of current developments in targeted immune cell and immune checkpoint inhibitory therapy.

## Background

Invasive breast cancer is the most frequent malignancy in women [1] and includes tumors with a wide range of histologic types, therapeutic response and overall prognosis. State-of-the-art imaging for early cancer detection and appropriate therapeutic strategies play a key role for the improvement of survival rates.

At present, dedicated breast MRI and ^18^F-FDG PET/CT offer highest diagnostic accuracy in the detection of breast cancer [2, 3] and metastases [4] respectively. With the increasing role of personalized medicine, however, the desire for molecular targeted approaches emerged, enabling high-specificity diagnostics and molecular targeted therapies with the appropriate molecular key target.

CXCR4 is a 7-transmembrane G-coupled receptor belonging to the chemokine receptor family and is expressed by a variety of cells during development and thereafter [5]. With its cognate ligand stromal cell-derived factor 1α (SDF-1α, named CXCL12) [6, 7], its main role in the hematopoietic system is to control stem cell retention and the homing of hematopoietic cells to the bone marrow and lymphoid organs [8]. In addition to these physiological roles, CXCR4 has been found to be overexpressed by various human cancers including breast cancer [5, 9–11]. In cancer, CXCR4 expression and its activation by its endogeneous ligand CXCL12 are key triggers for tumor growth and progression, invasiveness, and metastasis [7, 12]. High levels of CXCL12 in organs and tissues, such as lymph nodes, lung, liver, and bone/bone marrow (BM), are thought to direct the metastasis of CXCR4-expressing tumor cells [13]. Accordingly, the level of CXCR4 expression was shown to be higher in metastatic sites as compared to the primary tumors [10], and changes in CXCR4 signaling have been shown to significantly alter metastatic burden in animal models [14]. CXCR4 is not only expressed by cancer cells themselves, but also by tumor-infiltrating immune cells. Within the tumor microenvironment, the major CXCR4-expressing cells are B-lymphocytes and plasmacytoid dendritic cells, both potentially contributing to an immunosuppressive site permissive for tumor progression [15].

Recently, a radiolabeled CXCR4-ligand for PET (^68^Ga-Pentixafor) imaging has been developed by Wester et al. [16–18]. First in vivo studies in patients with hematological malignancies, glioblastoma and small cell lung cancer confirmed high tracer accumulation in these tumors with low background uptake and concomitant fast tracer clearance from non-target tissues [12, 19–21]. A recent publication of our group evaluated the feasibility of CXCR4-targeted PET in three patients with breast cancer amongst other solid cancers [21]. Although tracer uptake was low to moderate in all in these patients, we were curious to know if a wider spectrum of breast cancers (including different histological types) might show a more favorable tracer affinity.

The current study presents the first results on CXCR4-targeted PET imaging in a larger cohort of patients with primary and recurrent breast cancer.

## Methods

### Patients

This retrospective analysis included 13 patients (mean age 59, range 38–77) with histologically proven primary breast cancer (without previous therapy), four patients with recurrent breast cancer after treatment, and one patient with axillary lymph node metastasis but unknown primary examined between January 2014 and September 2016. Three patients from a previous published study were included in this cohort [21]. Patients underwent either ^68^Ga-Pentixafor PET/CT or ^68^Ga-Pentixafor PET/MR. Eight patients additionally received a diagnostic ^18^F-FDG PET/CT for staging purposes within 2 weeks after ^68^Ga-Pentixafor imaging. No therapy was performed between the two imaging modalities.

### Synthesis of ^68^Ga-Pentixafor

Synthesis of ^68^Ga-Pentixafor was performed on a fully automated, GMP-compliant procedure using GRP module (Scintomics GmbH, Germany) equipped with disposable single-use cassette kits following the previously described method [19, 21]. Prior to injection, tracer quality was assessed according to the standards of the European Pharmacopoeia available at www.edqm.eu.

### PET imaging protocol

Nine of 18 ^68^Ga-Pentixafor PET and all ^18^F-FDG PET scans were performed on a Sensation 64 Biograph PET/CT scanner (Siemens, Erlangen, Germany), whereas 9 of 18 ^68^Ga-Pentixafor scans were performed on a PET/MRI device (Siemens Biograph mMR, Siemens Medical Solutions, Germany). The CT-scan protocol included a low-dose CT (26 mAS, 120 kV, 5 mm slice thickness) from the base of the skull to the mid-thigh for attenuation correction followed by the PET scan and a diagnostic CT (240 mAS, 120 kV, 5 mm slice thickness) in the portal venous phase in case of ^18^F-FDG PET/CT scans. Injected activities for ^68^Ga-Pentixafor ranged from 177 to 223 MBq and for ^18^F-FDG from 201 to 349 MBq. PET acquisition was performed after a mean of 51 min post injection for ^68^Ga-Pentixafor and 75 min post injection for ^18^F-FDG PET/CT respectively. All PET/CT scans were acquired in 3D mode with an acquisition time of 3 min per bed position. Images were reconstructed by an attenuation-weighted ordered-subset expectation maximization algorithm (four iterations, eight subsets) followed by a post-reconstruction smoothing Gaussian filter (5 mm full-width at half-maximum). In PET/MR, a coronal 2-point Dixon 3D volumetric interpolated examination (VIBE) T1w sequence was performed for generation of attenuation maps as recently published [22]. In addition, diagnostic dedicated sequences dependent on the examined malignancy were performed. PET data was acquired simultaneously in three-dimensional mode with 3-min emission time per bed position.

### Image analysis

All ^68^Ga-Pentixafor PET/CT or PET/MR images were reviewed and interpreted by board-certified Nuclear Medicine physicians and by radiologists in consensus. Lesions were defined as visually detectable, if both reviewers were able to visually identify the lesions on the PET images. Semiquantitative SUV analysis with determination of SUVmax involved drawing region of interests (ROI) around the primary tumor in case of preoperative imaging and around the metastases, when detectable. Lesions below 10 mm in diameter were omitted in order to reduce partial volume effects. Analysis of ^18^F-FDG PET/CT in patients, who underwent both imaging modalities, was performed analogous to ^68^Ga-Pentixafor PET/CT. Maximum standardized uptake values (SUVmax) of ^18^F-FDG and to ^68^Ga-Pentixafor PET images were compared and correlated with each other. Referring to Drzezga et al., we assumed a relative difference of < 10% between SUV values of lesions obtained from PET/CT and PET/MR respectively and therefore did not differ between the SUV of the two modalities [22].

### Pathological analysis

Histology of primary breast lesions was obtained by image-guided biopsy in five cases and by open surgery in eight cases. The tissues were routinely fixed in 10% neutral buffered formalin, dehydrated, and cut into 2-μm-thick sections. Tumor classification according to the WHO 2012 criteria and assessment of the tumor biology (estrogen receptor; ER, progesterone receptor; PR, human epidermal growth factors receptor 2; HER2) was performed in routine pathological diagnostics. To further categorize all breast cancer samples according to the molecular subtypes, the proliferation index was assessed by immunohistochemistry using Ki67 (MIB1). In accordance to the St. Gallen guidelines [23], we used a local Ki67 cut off value of 25% to differentiate between luminal A and luminal B tumors. Tumors were categorized as follows: luminal A (ER+ and/or PR+, HER2/neu− and Ki67%<25%), luminal B HER2− (ER+ and/or PR+, HER2/neu− and Ki67%≥25%), luminal B HER2+ (ER+ and/or PR+, HER2/neu+), HER2/neu non-luminal (ER/PR−, HER2/neu+), and TNBC (ER/PR−, HER2/neu).

### Immunohistochemistry

Immunohistochemistry of biopsy and open surgery specimens was performed according to standard routine methods for Ki67 (Dako, M7240), ER status (DCS, EI 629C01), and PR status (DCS, PI 633C01) using the ultraVIEW DAB Detection Kit (all reagents from Ventana, Tucson, AZ). Briefly, the tissue sections were deparaffinized with EZ Prep at 75 °C and 76 °C, heat pretreated in Cell Conditioning 1 (CC1) for antigen retrieval at 76–100 °C, and then incubated with the primary antibody diluted in antibody diluent (ER 1:20, Ki67 and PR 1:50) for 20 min at 37 °C after inactivation of the endogenous peroxidase using a UV inhibitor for 4 min at 37 °C. The slides were incubated with a HRP Universal Multimer for 8 min. Antibody binding was detected using DAB as chromogen and counterstained with hematoxylin with subsequent bluing in bluing reagent.

CXCR4 immunohistochemistry was performed on a Dako Autostainer. After heat-induced antigen retrieval (target retrieval solution, pH 6, Dako, Glostrup, Denmark) for 20 min, unspecific protein and peroxidase binding was blocked with 3% hydrogen peroxide and 3% normal goat serum. A primary antibody against CXCR4 (clone UMB-2, Abcam, Cambridge, UK) was used and diluted 1:30 in antibody diluent (Dako, Glostrup, Denmark). For antibody detection, the Dako Envision-HRP rabbit labeled polymer (Dako, Glostrup, Denmark) was used. Antibody binding was visualized by diaminobenzidine (DAB) giving a brown precipitate (Medac Diagnostica, Wedel, Germany, BS04-500). Counterstaining was performed using hematoxylin.

Subsequent to immunohistochemical procedure, all slides were then dehydrated manually by alcohol washes of increasing concentration (70%, 96%, 100%) and xylene and coverslipped using Pertex® mounting medium (Histolab, Goeteborg, Sweden, 00801). CXCR4 expression was evaluated with respect to cell type (infiltrating immune cells vs. tumor cells), expression intensity, and percentage. Overall staining intensity for CXCR4 was scored into categories reaching from (-) (no staining) to (+++) (high staining intensity and frequency).

Ki67 staining was evaluated according to the recommendations of the International Ki67 in Breast Cancer Working group [24].

### Statistics

Statistical analysis was performed using MedCalc Version 10.2 (Mariakerke, Belgium). Correlation studies between SUVmax and PR status, ER status, and Ki67 proliferation index were performed with Spearman rank test. To test for significance between molecular subtypes and SUVmax, we used Kruskal-Wallis test.

## Results

### Patient characteristics

Thirteen of 18 patients were diagnosed with primary breast carcinoma without history of previous malignant breast lesions or therapy (Table 1). Lymph node (LN) metastases were present in 5 of 13 patients and distant metastases in 2 patients (one patient with liver metastases, one patient with bone metastases).

**Table 1:** CXCR4-targeted PET imaging with ^68^Ga-Pentixafor of primary breast cancer patients with corresponding age, tumor subtype, tumor grade (G), presence of lymph node (LN) metastases, or organ metastases, SUVmax of the primary tumor, and tumor-to-background (T/B) ratio

Patient #	Age	Subtype	Grade	LN MTS	Organ MTS	SUVmax	T/B ratio	Visible
1	61	NST	G2	−	−	3.2	2.9	+
2	58	NST	G3	+	+	4.3	3.9	+
3	49	ILC	G3	+	−	1.7	1.0	−
4	51	NST	G2	+	+	2.0	2.0	+
5	53	NST	G2	−	−	2.9	2.6	+
6	56	NST	G2	−	−	1.9	1.6	−
7	38	NST	G2	+	−	2.8	2.3	+
8	69	NST	G2	+	−	3.2	3.6	+
9	50	NST	G2	−	−	4.5	3.5	+
10	47	ILC	G2	−	+	1.8	1.0	−
11	40	NST	G2	−	−	1.8	1.0	−
12	67	NST	G2	−	−	2.9	1.6	+
13	48	NST	G3	−	−	5.7	3.8	+

In 9 of 13 patients, the primary tumor was visually detectable (last row)

*NST* invasive carcinoma of no special type, *ILC* invasive lobular carcinoma

Four of 18 patients demonstrated recurrent metastatic disease with three cases of isolated lymph node recurrence following primary breast cancer after an average of 36 months and one case of newly diagnosed liver metastases after 6 years following breast cancer (Table 2).

**Table 2:** CXCR4-targeted PET imaging of patients with recurrent breast cancer with corresponding clinical data

Patient #	Age	Type	Grade	SUVmax	T/B ratio	Visible
14	62	Nodal recurrence	G2	4.2	4.7	+
15	67	Nodal recurrence	G3	4.0	3.9	+
16	64	Hepatic recurrence	G3	3.8	2.8	+
17	71	Unknown primary	G2	4.5	3.3	+
18	71	Nodal recurrence	G2	2.0	1.5	+

All metastatic lesions were visually detectable on ^68^Ga-Pentixafor PET images

One patient exhibited lymph node metastases in the left axillary region, histologically verified as metastases from breast cancer, however, without evidence of the primary tumor in imaging studies.

### Results of ^68^Ga-Pentixafor PET imaging in primary breast cancer lesions

**Fig. 1:**
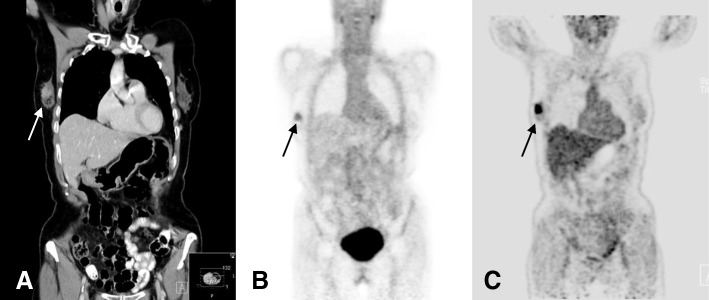
69-year-old patient with invasive ductal carcinoma (IDC) G2 with primary breast cancer prior to treatment. Coronal CT reconstruction shows contrast enhancement in a lesion with a diameter of 2.2 cm in the right breast (**a**). The tumor is visually detectable on ^68^Ga-Pentixafor PET with a corresponding SUVmax of 3.2 (**b**). On ^18^F-FDG PET/CT, the lesion demonstrates a significantly higher tracer uptake (SUVmax of 16.5) (**c**)

Primary breast cancers exhibited a mean SUVmax of 3.0 ranging between 1.7 and 4.5 and a mean T/B ratio of 2.4 (range 1 to 3.6). Nine of 13 tumors could be visually identified on ^68^Ga-Pentixafor PET images (Table 1; Fig. 1).

Highest SUVmax of 4.3 and 4.5 respectively were measured in two patients with invasive carcinoma of no special type (NST, G2 and G3 respectively). Lowest SUVmax between 1.7 and 1.9 were measured in two patients with invasive lobular carcinoma (ILC) and two patients with NST but without any hormone receptor and HER2 expression (triple negative breast cancer, TNBC) (Table 1). No significant difference was observed between SUVmax of metastasized and SUVmax of non-metastasized primary breast cancers (*p* = 0.37).

### Results of ^68^Ga-Pentixafor imaging in recurrent breast cancer lesions

**Fig. 2:**
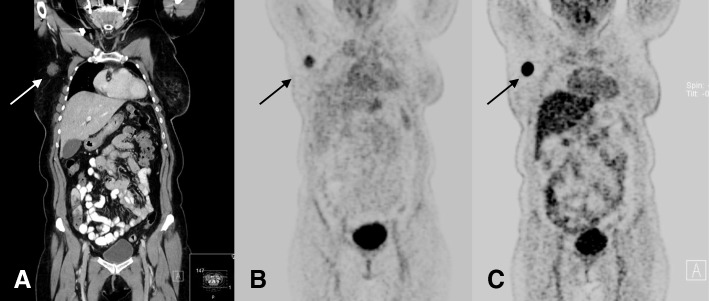
67-year-old patient with a nodal recurrence 22 months after treatment of a primary breast cancer. Coronal CT reconstruction shows a contrast enhancing lymph node metastasis with a diameter of 2.1 cm in the right axillary region (**a**). The lesion is visually detectable on ^68^Ga-Pentixafor PET with a corresponding SUVmax of 4.0 (**b**). On ^18^F-FDG PET/CT, the lesion shows a significantly higher tracer uptake (SUVmax of 24.4) (**c**)

Metastases of recurrent breast cancer were visually detectable in all five cases, exhibiting a mean SUVmax of 3.7 (range 2 to 4.5) and a mean T/B ratio of 3.2 (1.5 to 4.7) (Fig. 2). Highest SUVmax of 4.5 was observed in the patient with unknown primary cancer (Table 2).

### Immunohistochemistry

**Fig. 3:**
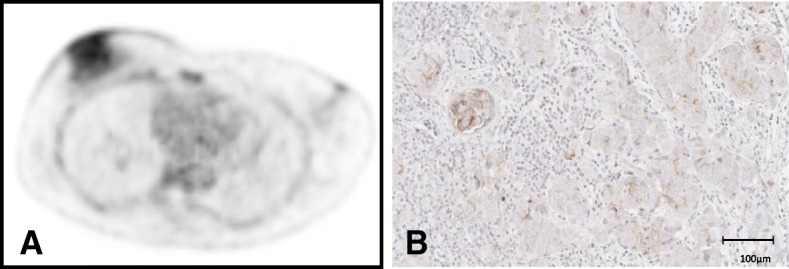
Example of a patient with primary breast cancer exhibiting moderate tracer uptake of the primary tumor on ^68^Ga-Pentixafor PET (**a**) and corresponding moderate CXCR4 expression on immunohistochemistry (**b**)

Immunohistochemical staining for CXCR4 of open surgery specimens was available in seven primary breast cancers (Table 3; Fig. 3). The two cases of invasive lobular carcinoma (ILC) were not or slightly infiltrated by immune cells. The inflammatory cells in the ILC cases did not express CXCR4. The invasive carcinomas of no special type (NST) displayed inter- and intratumoral heterogeneity of CXCR4 expression in the tumor cell-infiltrating immune cells and within the tumor cells (Table 3). To sum up the different expression frequency and intensity, an overall signal intensity of CXCR4 expression on the immunohistochemical level was applied. Five of seven samples demonstrated weak to moderate overall CXCR4 expression (+, ++), whereas two of seven samples did not demonstrate any CXCR4 expression at all (-). In five of seven samples, immunohistological overall staining intensity corresponded with tracer uptake during ^68^Ga-Pentixafor PET imaging. In the remaining two cases, immunohistochemistry did either not demonstrate any CXCR4 expression, although PET imaging revealed an SUVmax of 3.2 (patient #8, Table 1) or CXCR4 expression was rated as moderate despite a low SUVmax of 1.8 (patient #11, Table 1).

**Table 3:** Immunohistochemical assessment for CXCR4 in surgical specimens

Patient #	Subtype	Immune cells	Immune cell positivity (%)	Tumor cells	Tumor cell positivity (%)	Overall signal strength	SUVmax
3	ILC	+	0	−	0	−	1.7
5	NST	+++	40	++	15	++	2.9
6	NST	++	5	++	5	+	1.9
7	NST	++	10	+++	2	+	2.8
8	NST	+	5	+	0	−	3.2
10	ILC	−	0	+	20	+	1.8
11	NST	+++	20	+	20	++	1.8

Degree of immune cell infiltration was scored from (−) (no infiltration) to (+++) (strong infiltration). Staining intensity for tumor cells was scored from (−) (no expression) to (+++) (strong expression). Percentage amount of CXCR4-positive immune and tumor cells in the specimens (positivity) is shown in the third and fifth columns. The last columns show overall signal intensity and corresponding SUVmax during PET CXCR4-directed PET imaging respectively

Immunohistochemical analysis of ER status, PR status, HER2/neu status, Ki67 status, and tumor grade was performed routinely in all patients (Table 4). Statistical analysis did not reveal any significant correlation between SUVmax and ER status, PR status, or Ki67% (rho = 0.32, *p* = 0.26; rho = − 0.03, *p* = 0.9; rho = 0.31; *p* = 0.28). However, there was a weak association between SUVmax and molecular cancer subtypes (*p* = 0.08).

**Table 4:** Receptor expression profile of primary breast cancers including estrogen receptor (ER), progesterone receptor (PR) status, HER2/neu status, and Ki67 proliferation index

Patient #	ER (%)	PR (%)	HER2	KI67 (%)	Subtype
1	100	80	−	25	Luminal B HER2−
2	100	20	−	20	Luminal A
3	95	95	−	20	Luminal A
4	50	20	−	25	Luminal B HER2−
5	50	50	−	20	Luminal A
6	0	0	−	15	Basal like
7	95	50	−	15	Luminal A
8	100	30	−	30	Luminal B HER2−
9	85	85	+	20	Luminal B HER2+
10	95	95	−	10	Luminal A
11	1	0	−	50	Basal like
12	95	70	−	25	Luminal B HER2−
13	60	50	−	40	Luminal B HER2−

The molecular subtypes have been defined based on “surrogate markers”

### Comparison with ^18^F-FDG PET/CT

Clinical ^18^F-FDG PET/CT for clinical staging or re-staging was performed in 8 of 18 patients (4 patients with recurrent breast cancer, 4 patients with primary breast cancer). SUVmax obtained during ^18^F-FDG PET was higher in all cases compared to CXCR4-targeted PET (mean SUVmax of 16.2 vs. mean SUVmax of 3.6; *p* < 0.05) (Figs. 1 and 2) (Table 5).

**Table 5:** Additional FDG PET/CT was performed in 8 patients within 1 week after CXCR4-targeted PET

Patient #	Age	Type	Grade	SUVmax CXCR4	SUVmax FDG
2	58	Primary cancer	G3	4.5	10.1
8	69	Primary cancer	G2	3.2	16.5
9	50	Primary cancer	G2	4.5	9.7
11	40	Primary cancer	G2	1.8	2.6
14	62	Nodal recurrence	G2	4.5	17.5
15	67	Nodal recurrence	G3	4.0	24.4
17	71	CUP	G2	4.5	33.0
18	71	Nodal recurrence	G2	2.0	16.1

SUVmax obtained after ^18^F-FDG PET was higher in all examined cases compared to CXCR4-targeted PET

## Discussion

CXCR4 plays a key role in cancer pathogenesis, progression, and metastasis. Several in vitro studies have previously demonstrated that CXCR4 is overexpressed in breast cancer and might be therefore an interesting target for diagnostic and therapeutic approaches [10, 14, 25].

In this study, we have demonstrated that in vivo imaging of CXCR4 using the PET probe ^68^Ga-Pentixafor is feasible in patients with primary and recurrent breast cancer. Intensity of tracer accumulation however was significantly lower in all examined patients when compared to ^18^F-FDG PET, confirming our initial observations in a previous publication that solid tumors in general tend to show lower ^68^Ga-Pentixafor accumulation in comparison to ^18^F-FDG PET [21].

Moreover, CXCR4-targeted tracer accumulation in the breast tumors varied significantly between patients. Histological tumor type seems to be one of the factors accounting for this heterogeneity. While most NST were visually detectable on ^68^Ga-Pentixafor PET, both cases of ILC in our study cohort were visually negative and showed low staining intensity in immunohistochemistry. These results go in line with previous reports that demonstrated a significantly lower staining intensity for ILC compared to NST [26].

The remaining two visually undetectable cancers were comprised of TNBC with low and moderate CXCR4 staining intensity respectively in immunohistochemistry. This is in particular interesting, since previous in vitro studies have reported high CXCR4 expression in this aggressive cancer subtype.

One of the factors accounting for this inconsistency might be predominantly cytoplasmatic CXCR4 expression in TNBC that has been reported in previous literature [27]. Chemokine receptors are, together with their ligands, internalized after binding as an important feature of the chemokine functions [28]. Previous studies reported that breast cancer cells exhibit different ratios of membrane localized versus cytoplasmatic CXCR4. A study by Blot et al. for example demonstrated membrane localized CXCR4 in only 25% of breast tumors compared to 81% with diffuse cytoplasmatic CXCR4 using immunostaining [29].

The amount of cytoplasmatic CXCR4 seems to be dependent on simultaneous overexpression of CXCL12 leading to enhanced CXCR4/CXCL12 internalization.

^68^Ga-Pentixafor, however, like most peptide-tracers are dependent on membrane-localized receptor expression and are thus unable to pass the membrane. As a consequence, cytoplasmatic CXCR4 does not contribute to signal intensity in PET imaging, resulting in lower overall signal intensity of breast cancer cells than possibly assumed by in vitro examinations.

In particular, several conflicting results between in vivo and in vitro imaging might be accounted to the observation that the aforementioned dynamic equilibrium between CXCR4 in the cytoplasm and on the plasma membrane modulates the target density at the cell membrane [25, 27, 30]. As a consequence, assessment of CXCR4 expression by immunohistochemistry that may include cytoplasmatic CXCR4 not necessarily correlates with PET imaging signal strength.

Our immunohistochemical findings support this hypothesis: while immunohistochemical CXCR4 expression corresponded with PET tracer accumulation in five of seven primary breast tumors, one of the aforementioned TNBC was not visible in PET imaging despite moderate CXCR4 staining intensity.

Of note, one patient with an NST exhibiting an SUVmax of 3.2 on CXCR4 PET imaging did not exhibit any immunohistochemical CXCR4 expression. We assume that tumor heterogeneity might be the cause for this discrepancy. Referring to this last case, it should be emphasized that cross-validation of receptor expression by means of immunohistochemistry and PET imaging is often difficult and hampered by tumor heterogeneity and less-representative sample collection for IHC. Results should be therefore interpreted with care. In particular, for CXCR4 imaging, intratumoral hypoxia might have a great influence, as it is known that CXCR4 is strongly upregulated in hypoxic tumor regions [31]. Additionally, the CXCR4/CXCL12 axis is a volatile system, part of a large network of the extracellular matrix/tumor cell microenvironment [15] with high spatiotemporal differences. Thus, in vivo imaging and immunohistochemistry from resection specimen might also differ due to a timely delay between imaging and resection.

A correlation between tracer accumulation and single prognostic factors including molecular subtype, PR status, ER status, tumor grade, or Ki67% was not observed in this study. Most previous in vitro studies were in concordance with our results. Kishima et al. examined the expression of CXCR4 mRNA using qPCR in breast cancer patients but failed to find any correlation to aforementioned prognostic factors [32]. Similar results were observed in a meta-analysis by Zhang et al. [33]. It is noteworthy that although different studies failed to find a correlation between ER status and CXCR4 expression, CXCR4 signaling is supposed to promote ER-positive breast cancer to a therapy-resistant, estrogen-independent phenotype [34]. CXCR4 in vivo imaging in these cases might have a benefit to provide longitudinal spatiotemporal information on tumor progression/metastasis during targeted estrogen deprivation therapy.

Further areas of interest for CXCR4-directed imaging might include HER2-positive breast cancers. Li et al. demonstrated a significant correlation between CXCR4 and HER2 expression using immunohistochemistry, supporting the idea that HER2 overexpression enhances CXCR4 expression and that both markers serve as a predictor for poor overall patient survival [35]. Our cohort included only one patient with HER2-positive breast cancer; therefore, we could not perform any correlation studies on this level; interestingly, however, this tumor showed high tracer accumulation and thus supporting the aforementioned hypothesis.

In case of recurrent breast cancer, metastases were visually detectable in all cases and tended to exhibit higher tracer positivity compared to primary breast cancer, however, not reaching statistical significance (*p* = 0.18). These results might reflect higher CXCR4 expression in recurrent, i.e., highly aggressive tumors [11, 14, 33], as described in previous literature.

We are aware that major limitations of the current study include a small patient cohort and the use of two imaging modalities (PET/CT and PET/MRI respectively), resulting in a limited interpretation of the statistical analysis. However, in this preliminary study, we rather intended to provide a first glimpse of imaging characteristics of ^68^Ga-Pentixafor in different breast cancer types including histological types, molecular subtypes, and primary/recurrent cancers. The heterogeneity of the current imaging results seems to reflect the highly complex biological interactions of CXCR4 that are only partially understood. Besides the aforementioned factors including dynamic equilibrium between CXCR4 in the cytoplasm and on the plasma membrane, various other interactions probably play an important role in CXCR4 imaging, aggravating interpretation of imaging results. Such interactions might include upregulation of CXCR4 by different factors (e.g., estradiol), tumor cell-stromal cell interactions, recruitment of CXCR4-positive immune cells to tumor sites inducing local inflammatory response [36].

Future CXCR4 imaging studies should investigate whether this modality might be useful in more specific applications. For example, CXCL12/CXCR4 axis is also of high interest for immunotherapeutical approaches due to its crucial role in tumor initiation and progression; therefore, possible future applications might include patient selection and therapy monitoring for targeted therapies. CXCR4-targeting PET tracers with higher affinity are currently under development (personal communication Prof. H.J. Wester) and may enhance image quality in the future.

## Conclusions

In conclusion, CXCR4-targeted PET imaging of primary and recurrent breast cancer is feasible. CXCR4-targeted tracer accumulation in tumor tissue is heterogeneous and seems to be, inter alia, dependent on histological subtype.

A correlation between intensity of tracer uptake in CXCR4-directed PET imaging and prognostic factors ER, PR, proliferation index, tumor grade, and tumor biology was not observed. Moreover, tracer accumulation in tumor tissue was significantly lower compared to ^18^F-FDG PET imaging. Consequently, based on this pilot study with a small patient cohort, CXCR4-targeted PET failed to clearly demonstrate its usefulness for imaging of breast cancer.

Further studies are necessary to understand the complex biology of CXCR4 and to accordingly interpret imaging results.
